# Supplementary material for: The Ability of Biodegradable Thermosensitive Hydrogel Composite Calcium-Silicon-Based Bioactive Bone Cement in Promoting Osteogenesis and Repairing Rabbit Distal Femoral Defects
Source: Polymers (Basel). 2022 Sep 15;14(18):3852. doi: 10.3390/polym14183852 (PMC9503108; doi:10.3390/polym14183852)
Supplement: Supplementary file 1 [file polymers-14-03852-s001.zip › polymers-1722342-supplementary.pdf]

polymers

Article

The Ability of Biodegradable Thermosensitive Hydrogel Composite Calcium-Silicon-Based Bioactive Bone Cement in Promoting Osteogenesis and Repairing Rabbit Distal Femoral Defects

Chao Guo [1,†], Junqiang Qi [1,†], Jia Liu [1], Haotian Wang [1], Yifei Liu [1], Yingying Feng [2] and Guohua Xu [1,*]

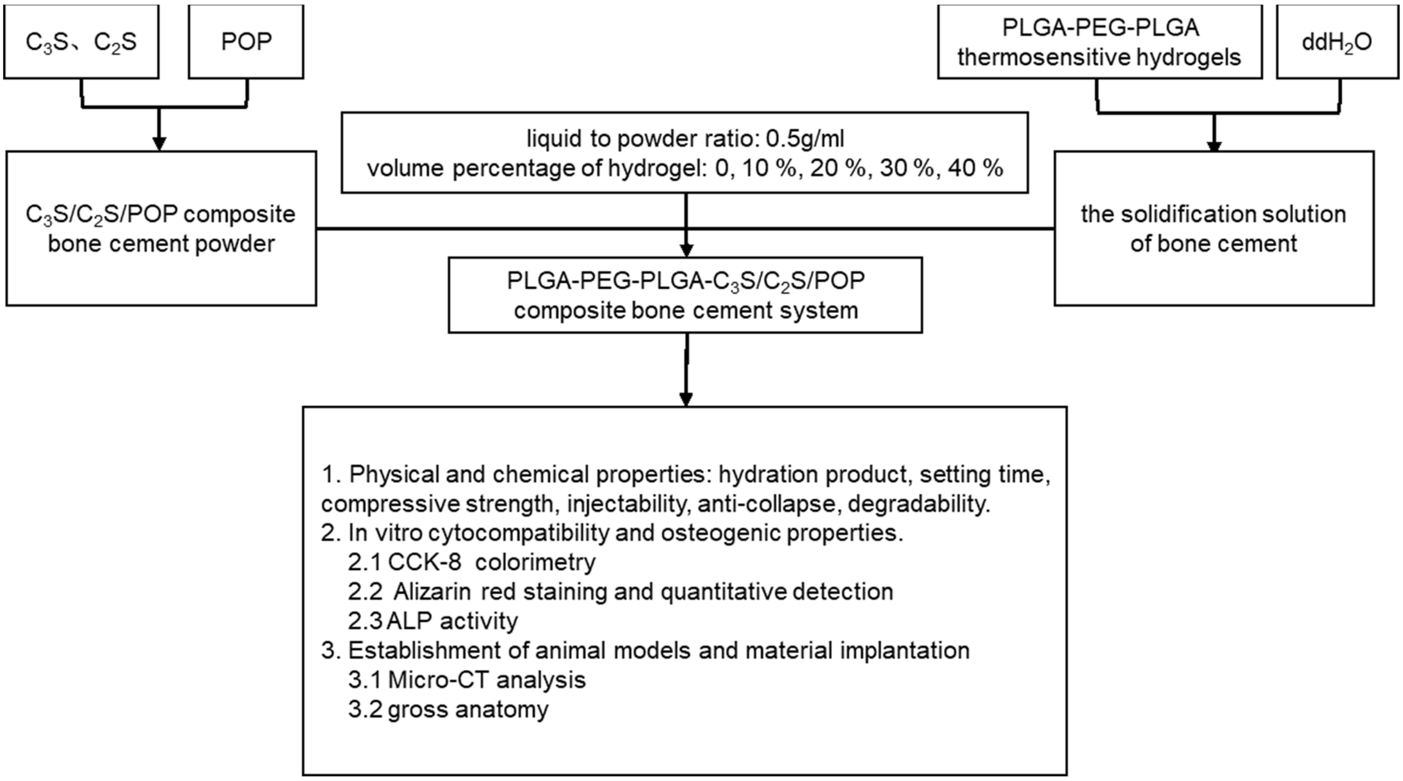

Figure S1. The experimental flow chat.
